# Spatial Effect Analysis of Health Expenditure and Health Output in China From 2011 to 2018

**DOI:** 10.3389/fpubh.2022.794177

**Published:** 2022-04-04

**Authors:** Penghui Xu, Xicang Zhao, Haili Li, Shi Guo

**Affiliations:** ^1^School of Management, Jiangsu University, Zhenjiang, China; ^2^School of Humanities and Management, Wannan Medical College, Wuhu, China

**Keywords:** health expenditure, health output, spatial lag model, spatial Durbin model, spatial spillover effect

## Abstract

**Objective:**

The objective of this study is to study the spatial effects of health expenditure and health output in China.

**Methods:**

Using the spatial panel data of 31 provinces in China from 2011 to 2018, the spatial weight matrix was introduced to analyze the spatial correlation, and the spatial Durbin model (SDM) was used to investigate the health output effect of health expenditure.

**Results:**

Excluding the number of doctors per thousand, the provincial health expenditure, the number of beds per thousand population, and per capita education level had a positive impact on the regional health output. The health effect of China's health inputs showed a spatial spillover effect.

**Conclusion:**

Due to the significant spatial effect, the health output of 31 provinces in China benefits not only from the local health inputs, but also from the health inputs of neighboring provinces.

**Suggestions:**

This article puts forward some suggestions based on the conclusion: China should strengthen the health cooperation among neighboring provinces, promote the free flow of various health factors among provinces, make full use of the spillover and interdependence of health investment among provinces, and improve the medical policy environment in China.

## Introduction

Under the concept of the neoclassical growth model, human capital, particularly good human health, is an imperative factor for attaining the desired economic growth and development of any country (1). Besides, based on Grossman's human capital model, health increases the human capital by making more time for working and increasing utility (2). The promotion of health is substantial because health is vital, a fundamental right (3). At present, health is the center of sustainable development, and increasing the individuals' health level is one of the most important policies of countries. Therefore, investment in health is very important.

Health expenditures contain all the expenditures which are used for preparing and improving the individuals' health (4). The concept of health expenditures is different from one country to another. In China, health expenditure refers to the total amount of funds consumed by the whole society for medical and health services in a certain period of time (usually 1 year) in a country or region, which includes the total monetary amount of living labor and materialized labor consumed in providing healthcare services (5). Health expenditure can not only comprehensively reflect the basic situation of a country and region, such as total health investment, resource allocation efficiency, and population medical burden, but also an important macroindicator of health economy to scientifically evaluate whether the investment is sufficient, whether the financing is fair, and whether the allocation is effective (6). From 2011 to 2018, China's health expenditure maintained a rapid growth, with an increase of about 242.84% in 8 years, from 2,434.591 billion Yuan in 2011 to 5,912.191 billion Yuan in 2018; the infant mortality rate decreased from 12.1% in 2011 to 3.9% in 2018, a decrease of 67.76%.

Over the past few decades, scholars and researchers have paid more attention to evaluate the link between health expenditure and health outcomes. Relevant studies can be mainly summarized in two aspects. Some studies showed that health expenditure leads to better health outcomes, whereas others have reported an insignificant effect. For instance, Musgrove (7), Filmer and Pritchett (8), and Fayissa and Gutema (9) both concluded that health expenditure is not a crucial determinant of health outcomes. Furthermore, Fayissa and Gutema (9) reported a strong negative effect of increases in health expenditure on life expectancy at birth, and Gupta et al. (10) reported that the effect of public health expenditure on health outcomes is weak. Kiross et al. (11) used panel data from World Bank Development Indictors (WDI) from 2000 to 2015 covering 46 countries in sub-Saharan Africa, used the random effects model, and found that public and external healthcare expenditure was significantly negatively correlated with health output. Based on dynamic panel system GMM model, Shen et al. (12) suggest that private health expenditure had no significant impact on health output.

On the other hand, studies by Anyanwu and Ehijakpor (13), Kamiya (14), and Novignon et al. (15) have reported a positive effect of health expenditure on health outcomes. Whereas these studies have reported a positive effect of health expenditure on health outcomes, the conclusions from these studies have differed. Bein et al. (16) and Raeesi et al. (17) suggest that health outcomes improved with the increase of public and private healthcare expenditure. Zhang et al. (18) and Mao et al. (19) both propose an increase in public health expenditure to improve health outcomes. Novignon et al. (15) suggest that the effect of public health expenditure is stronger than that of private. However, Rahman et al. (20) suggest that the effect of private health expenditure is greater than that of public health expenditure. Zhao et al. (21) adopted the vector error correction model and found that the total health expenditure in China had a significant improvement effect on health output.

Based on our review of the literature, first, we identify that there is no consistent conclusion on the effects of health expenditure on health outcomes. Second, most scholars have used the random and fixed effect models, cointegration test, and the vector error correction model to investigate the effects of health expenditure on health outcomes, which is likely to cause errors or biasness in estimating and analyzing the process, and they fail to consider the spatial correlation of health outputs and whether there are spatial spillover effects of health expenditure. Based on the spatial panel data of 31 Chinese provinces obtained from 2011 to 2018, we employ the spatial Durbin model (SDM) to analyze the effects of health expenditure on health outcomes and finally puts forward some conclusions and suggestions based on the deepening of cognition of healthy China.

## Research Design

### Model Setting and Variable Selection

Based on the aim of this study, we included the relationship between health expenditure and health development into the system subframework. Based on the health production function Grossman (2) and the definition of “social determinants of health” by the World Health Organization (22), a macrohealth production function model is established as follows:


(1) $$\begin{array}{l} \text{lnHealt}h_{it}=\alpha_{0}+\beta_{1}\text{lnH}I_{it}+\beta_{2}\text{lnEd}u_{it}+\beta_{3}\text{lnBe}d_{it}+\beta_{4}\text{lnDo}c_{it}+\varepsilon_{it} \end{array}$$

where i is region, t represents year, α denotes constant, ε represents the random error term, and β_1_−β_4_ represent the coefficient matrix of each variable, respectively, and measure the influence degree of each variable on the explained variable. As the dependent variable, we use health to express the health output. Other variables are independent variables. The specific description and sources of the dependent and independent variables are as follows:

(1) Health output (health): Based on our review of the literature, we found that the measurement indicators of health output generally focus on life expectancy per capita, infant mortality, maternal mortality, mortality, mortality of children under the age of 5 years, etc. Considering the availability of data and the authority of indicators, according to the research of Yang and Lu (23), we adopt the provincial annual population mortality (%) indicator to measure health, which is a negative indicator, that is, the higher the value is, the lower the regional health level is. Failure to forward the reverse index will lead to the deviation in the reliability of the analysis results, which will have an adverse impact on the correctness of the decision (24). Therefore, we use the reciprocal method to forward the data of the reverse index based on SPSS20.0 software.
(2) The provincial health expenditure (HI) denotes health expenditure and health input of each province. The health expenditure refers to the total amount of funds consumed by the whole society for medical and health services in 1 year in each province, which includes the total monetary amount of living labor and materialized labor consumed in providing healthcare services (5).
(3) The number of beds per thousand (Bed) refers to the number of medical beds per thousand people, which indicates the medical facilities and service level of each region. The number of doctors per thousand has a significant positive impact on health output (13), and the increase in the number of beds will promote residents' demand for health, so as to improve their health status and improve their health level (25).
(4) Per capita education level (Edu) refers to the average level of education in a region. According to Grossman's research, good education plays an important role in obtaining job opportunities, obtaining nutrition, forming a good lifestyle, and efficient use of drugs (2). It is of great significance to improve the quality of life and health level. That is, per capita education level has a significant positive impact on health output (26).
(5) The number of doctors per thousand (Doc) refers to the number of health technical personnel per thousand people, representing the medical technical personnel in each region, reflecting the level of health services in a region. In the context of shortage of health human resources, increasing human input can obtain higher health output (27). Establishing dynamic panel data from the macrolevel and using GMM estimation method, Shen et al. (28) found that the number of doctors per thousand people can significantly improve the health level.

### Data Sources

In accordance with the consistency, validity, and availability of data, statistical data are collected from 31 provinces during 2011 and 2018, and the data sources include the China Statistics Yearbook (2012–2020), the China Health Statistics Yearbook (2012), China Health and Family Planning Statistics Yearbook (2013–2017), the China Health Statistics Yearbook (2018–2020), and wind database. All variables are adopted as their natural logarithm for processing potential heteroscedasticity, as listed in Table 1.

**Table 1:** Descriptive statistics of variables.

**Variable**	**Description**	**Mean**	**Std. dev**.	**Min**	**Max**
Health	The provincial annual population mortality	0.5407	0.2392	0	1
HI	The provincial health expenditure	1286.31	979.3121	63.97	5198.69
Doc	The number of doctors per thousand	5.9034	1.6025	2.68	15.46
Bed	The number of beds per thousand	4.9939	0.9736	2.77	7.55
Edu	Per capita education level	9.0162	1.1308	4.2219	12.6754

## Method

### Spatial Econometric Models

To examine and measure the possible spatial effects, the spatial lag model (SLM), the spatial error model (SEM), and the SDM have been utilized, including that SLM includes the regression model of spatial dependence through the addition of a lagged dependent variable; SEM believes that the spatial correlation of variables may ignore the error term of the regression model through the addition of the dependent variable, whereas SDM not only reveals spatial spillover effect of dependent variable in adjacent regions, but also captures influence of independent variables in adjacent regions on their own dependent variables. It is a common model for empirical test of spatial spillover effect (29). These three spatial econometrics models were constructed as follows:

SLM:


(2) $$\begin{array}{l} \text{lnHealt}h_{it}=\rho W\text{lnHealt}h_{it}+\beta_{1}\text{lnH}I_{it}+\beta_{2}\text{lnDo}c_{it}+\beta_{3}\text{InBe}d_{it}+\beta_{4}\text{lnEd}u_{it}\\ \text{+}\mu_{i}+\lambda_{t}+\varepsilon_{it},\varepsilon_{it}\sim N\left(0,\sigma^{2}I_{\text{n}}\right) \end{array}$$

SEM:


(3) $$\begin{array}{l} \text{lnHealt}h_{it}=\beta_{1}\text{lnH}I_{it}+\beta_{2}\text{lnDo}c_{it}+\beta_{3}\text{InBe}d_{it}+\beta_{4}\text{lnEd}u_{it}\\ \text{+}\mu_{i}+\lambda_{t}+\phi_{it}\\ \phi_{it}=\eta \Sigma_{j=1}^{N}W_{i\text{j}}\phi_{it}+\varepsilon_{it},\varepsilon_{it}\sim N\left(0,\sigma^{2}I_{\text{n}}\right) \end{array}$$

SDM:


(4) $$\begin{array}{l} \text{lnHealt}h_{it}=\rho W\text{lnHealt}h_{it}+\beta_{1}\text{lnH}I_{it}+\beta_{2}\text{lnDo}c_{it}+\beta_{3}\text{InBe}d_{it}+\beta_{4}\text{lnEd}u_{it}\\ \text{+}W\left(\theta_{1}\text{lnH}I_{it}+\theta_{2}\text{lnDo}c_{it}+\theta_{3}\text{InBe}d_{it}+\theta_{4}\text{lnEd}u_{it}\right)\text{+}\mu_{i}+\lambda_{t}+\varepsilon_{it}\\ \varepsilon_{it}\sim N\left(0,\sigma^{2}I_{\text{n}}\right) \end{array}$$

Where β denotes the direct coefficient of the independent variable, which indicates the impact exerted by the independent variable on the dependent variable. θ denotes the space lag coefficient of the independent variable, which indicates the impact exerted by the independent variable in surrounding cities on the dependent variable in the local city. ρ denotes the spatial autoregressive coefficient, which indicates the degree of dependent variable's spatial dependence. λ_*t*_ stands for the time fixed effect, μ_*i*_ refers to the space fixed effect. *W* denotes the economic distance weight matrix (W). *Wy, Wx* denote the spatial lag terms of dependent variable and independent variable, which allows us to analyze the spillover effects of independent variables. ε_*it*_ denotes a random error vector, satisfying $\varepsilon_{it}\sim \;N\left(0,\sigma_{it}^{2}\right)$. η measures the impact intensity of the error of the dependent variable in neighborhood cities. i is the 31 province units in China, and t represents year.

To judge which spatial econometric model is more appropriate, Elhorst (29) proposed a test method: to test and sum the two hypotheses of SDM panel model through Wald test and LR test based on *H*_0_:θ = 0 and *H*_0_:θ+ρβ = 0. If both assumptions are rejected, SDM panel model should be used; If θ = 0, and LM test and robust LM test show that the dependent variables have spatial correlation, SDM converts into a SLM; If θ+ρβ = 0, and LM test and robust LM test show that the residual has spatial autocorrelation, SDM simplifies into a SEM. The specific econometric model is Equations (2)–(4). All variables are presented in logarithmic forms to eliminate possible heteroscedasticity.

### Spatial Weight Matrix

Generally, the spatial weight matrix based on the economic correlation has also been widely utilized in spatial econometrics (30). Therefore, we construct and employ the economic distance weight matrix (W) to investigate the effects of health expenditure and health output. The spatial weight matrix was constructed as follows:


(5) $$\begin{array}{l} W=\{\begin{array}{ll} \frac{1}{\left|\bar{GDP_{i}}-\bar{GDP_{j}}\right|}, & \begin{array}{cc} if & i\neq j \end{array}\\ 0, & \begin{array}{cc} if & i=j \end{array} \end{array} \end{array}$$

where $\bar{GDP_{i}}$, $\bar{GDP_{\text{j}}}$ refer to the annual average GDP of province i and j during the research period 2011–2018, respectively. Here, we adopt the row normalization to ensure the rows sum to 1, and their diagonal elements are set to 0.

### Data Description of Space Metering Panel

The global Moran's I index is used to examine and measure the spatial autocorrelation and spatial heterogeneity. The equation for calculating global Moran'I is defined as follows:


(6) Moran'I = ∑i=1n∑j=1nWij(Yi−Y¯)(Yj−Y¯)S2∑i=1n∑j=1nWij

among them, $S^{2}=\frac{1}{n}\overset{n}{\underset{i=1}{\sum }}\left(Y_{i}-Ȳ\right)$, $Ȳ=\frac{1}{n}\overset{n}{\underset{i=1}{\sum }}Y_{i}$, *Y*_*i*_ represents the observed value of the region, *n* denotes the total number of provinces, and *W*_*ij*_ denotes the economic distance weight matrix.

The values of global Moran's I range from −1 to 1. The value of global Moran's I is greater than zero that means there has positive spatial correlation, with value less than zero that means there has negative spatial correlation, with value equal to zero that means there has no spatial correlation.

As shown in Table 2, the Morans'I index of each variable was significantly positive, which indicates that health, HI, Bed, Edu, and Doc in China's 31 provinces have spatial autocorrelation. Therefore, it is appropriate for us to adopt the empirical method of spatial measurement.

**Table 2:** Moran's I index test results of core variables.

**Year**	**Health**		**HI**		**Doc**		**Bed**		**HR**	
	**Moran's I**	**Z value**	**Moran's I**	**Z value**	**Moran's I**	**Z value**	**Moran's I**	**Z value**	**Moran's I**	**Z value**
2011	0.151*	1.579	0.128**	1.933	0.260***	4.144	0.144**	1.629	0.302***	3.106
2012	0.171**	1.753	0.131**	1.980	0.155***	2.562	0.138**	1.488	0.307***	3.193
2013	0.128*	1.380	0.137**	2.055	0.266***	4.250	0.179**	1.834	0.287***	3.184
2014	0.201**	2.006	0.145**	2.157	0.114**	1.990	0.215**	2.120	0.255***	2.923
2015	0.123*	1.331	0.150**	2.217	0.105**	1.892	0.244***	2.352	0.331***	3.407
2016	0.172**	1.750	0.153**	2.261	0.097**	1.755	0.252***	2.422	0.328***	3.388
2017	0.197**	1.954	0.156**	2.300	0.079*	1.515	0.306***	2.877	0.301***	3.108
2018	0.181**	1.832	0.160**	2.346	0.072*	1.454	0.328***	3.068	0.304***	3.141

***, **, * Represent significance levels of 1, 5, and 10%, respectively.

### Model Test

First, the use of two Lagrange multiplier tests (i.e., LM_test for no spatial lag and Robust LM_test for no spatial lag, and LM_test for no spatial error and Robust LM_test for no spatial error) determines whether the spatial lag effect or the spatial error effect is significant (31). If one LM test shows a significant effect whereas the other effect is not significant, this study should adopt the significant form spatial effect model. If the LM test results show that the two effects are significant or are not significant simultaneously, this study should adopt the SDM, and by the Wald or likelihood ratio (LR) test could determine whether the SDM can be simplified into the SLM or SEM. Through the Hausman test and LR joint significance test (i.e., space fixed effect or time fixed effect), this study could determine whether the spatial econometric model should adopt pool fixed effect, space fixed effect, time fixed effect, or space-and-time fixed effect (32).

Because the SDM has jointly captured the influence of spatial lag dependent variable and spatial lag explanatory variables, Equations 2–4 may demonstrate the endogenous problem, which violates the classical assumptions of ordinary least square (OLS) method. Hence, LeSage et al. (33) put forward the maximum likelihood (ML) method to solve the endogenous problem effectively and provided the theoretical framework to estimate or analyze the spatial lag values of both dependent and independent variables (direct and indirect effects). The specific derivation processes are rewritten as:


(7) $$\begin{array}{l} y={\left(I-\rho W\right)}^{-1}\left(X\beta +\theta WX+\mu_{i}+\varepsilon_{it}\right) \end{array}$$

(8) $$\begin{array}{l} {\left(I-\rho W\right)}^{-1}=I+\rho W+\rho^{2}W^{2}++\rho^{3}W^{3}+\cdots \end{array}$$

(9) $$\begin{array}{l} \partial y_{i}/\partial x_{ir}={\left(I-\rho W\right)}^{-1}(I\beta_{r}+{\left(W\right)}_{ii}\theta_{r}), \end{array}$$

for all i and for all *r*,


(10) $$\begin{array}{l} \partial y_{i}/\partial x_{jr}={\left(I-\rho W\right)}^{-1}(I\beta_{r}+{\left(W\right)}_{ii}\theta_{r}), \end{array}$$

for all *i*≠*j* and for all *r*,

where y is the health, ρ denotes the spatial autocorrelation coefficient, W stands for non-negative spatial weight matrix, X represents the independent variables, and β and θ are the spatial regressive coefficients. Wy and WX denote the spatial lag terms of dependent variable and independent variable, which allows us to analyze the spillover effects of independent variables. *I* represents an *N*×1 unit matrix, N is the number of the provinces, (*I*−ρ*W*)^−1^ stands for spatial Leontief inverse matrix, ∂*y*_*i*_/∂*x*_*ir*_ denotes the direct effect, ∂*y*_*i*_/∂*x*_*jr*_ refers to the indirect effect. indicates the coefficient of the rth independent variable, and θ_*r*_ denotes the coefficient of the spatial lag of the rth independent variable. Referring to the methods presented by Elhorst (32), we use MATLAB to estimate the magnitude and sign of the direct and indirect effects in the SDM model.

## Results

Before performing the spatial econometric regression analysis, it is necessary to perform Lagrange multiplier (LM) test on the model to further determine whether the model has the spatial correlation. The original assumption of the LM test is that there is no spatial lag term and spatial error term. In Table 3, both the LM test and the robust LM test reject the null hypothesis at the significance level of 10%. Therefore, the introduction of space weight matrix is necessary and effective.

**Table 3:** The result of LM test and Hansman test.

**Variable**	**Statistics**	***p***
LM_lag	3.0409*	0.071
LM_error	4.1388**	0.040
Robust LM_lag	3.4522*	0.063
Robust LM_error	4.2861**	0.038
Hausman	60.790***	0.001

***, **, * Represent significance levels of 1, 5, and 10%, respectively.

To make the model more robust, the Hausman test was performed to determine whether the model should adopt fixed or random effects. Then, the Wald and likelihood ratio (LR) tests were conducted to determine whether the optimal SDM model should degenerate into the SLM or SEM models. The Hausman test results reject the random effects model hypothesis at the 1% significance level. Furthermore, in Table 4, both the Wald and LR test results reject the hypothesis that the SDM degenerates at the 1% significance level, which means that both null hypotheses are rejected; Besides, the dynamic SDM model was used to estimate, and the results were not ideal. As a result, the SDM model was selected in this study, and no dynamic models were pursued.

**Table 4:** Wald test and LR test of spatial panel model.

** 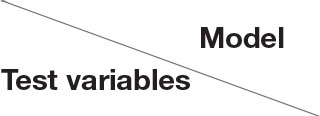 **	**Space fixed effect**	**Time fixed effect**	**Space time fixed effect**
Wald_spatial_lag	2.1770	18.3616***	19.1156***
	*P* = 0.7032	*P* = 0.0048	*P* = 0.0045
LR_spatial_lag	2.4562	15.4859***	17.1050***
	*P* = 0.6525	*P* = 0.0098	0.0046
Wald_spatial_error	18.8924***	17.2244***	22.0234***
	*P* = 0.0028	*P* = 0.0051	*P* = 0.0033
LR_spatial_error	17.1828***	18.3018***	19.1228***
	*P* = 0.0043	*P* = 0.0032	*P* = 0.0034

*** Represent the significance levels of 1%.

Elhorst believes that the panel data model with spatial lag-dependent variables is more reasonable than the R2 indicator (29). Through comparison of corrected goodness-of-fit (corrected R2) of the models, the values of no fixed effect, spatial fixed effect model, time fixed effect model, and double fixed model are 0.3544, 0.3591, 0.6544, and 0.5307, respectively. The time fixed effect model is obviously larger than those of other three models. The log-likelihood (log-L) values of time fixed effect model are significantly larger than those of other three models. Except LnPGDP variable, the coefficients of LnHI, LnDoc, LnBed, and LnEdu are positive and all significant at 5% significance level. To sum up, this study will select the time fixed effect model as a spatial econometric model to study the impact of health expenditure on health output in China.

From the results of the dynamic SDM regression in column (3) of Table 5, we analyze the impact of spatial effects on health. We find that the estimated parameter of LnHI is 0.0660 and significant at 1% significance level. LnHI has played a significant role in promoting health, and it is imperative to increase health expenditure. This also proves that China has been actively engaged in “Healthy China.” The coefficient of W^*^LnHI is 0.0625 and significant, which means that LnHI in local provinces is conducive to improve health in adjacent provinces. At present, LnHI has a strong spatial spillover effect in the geographical adjacent space. The coefficient of LnDoc is negative and significant. The coefficient of W^*^LnDoc is 0.1324 and significant, which means that LnDoc in local provinces is conducive to improve health in adjacent provinces. It indicates that when emphasis is attached to number of health technicians, per thousand people can achieve the goal of improving health. The coefficient of LnBed is 0.1317 and significant at 1% level. LnBed has played a significant role in promoting health, and it is imperative to increase the number of beds per thousand people. This also proves that China has been actively engaged in “Healthy China.” The coefficient of W^*^LnBed is 0.0966 and significant, which means that LnBed in local provinces is conducive to improve health in adjacent provinces. LnBed has a strong spatial spillover effect in the geographical adjacent space. The coefficient of LnEdu is positive and significant. LnEdu has played a significant role in promoting health in local provinces. The coefficient of W^*^LnEdu is −0.1152, which is significant at 10% significance level, which means that LnEdu in local provinces is not conducive to improve health in adjacent provinces. To further explore the spatial effects of regression coefficients in the spatial econometric model, we estimate the direct and indirect effects caused by changes in health inputs and their sum effects. Table 6 shows the decomposition results of the SDM.

**Table 5:** Setting results of four effects in SDM panel model.

** 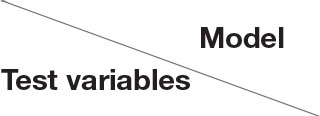 **	**1**	**2**	**3**	**4**
	**No fixed effect**	**Space fixed effect**	**Time fixed effect**	**Double-fixed**
LnHI	0.0260	0.0100	0.0660***	0.0620***
	(0.5348)	(0.5317)	(4.1186)	(3.9044)
LnDoc	−0.0229*	−0.0226*	−0.1057***	−0.1048***
	(−1.7325)	(−1.6013)	(−8.6248)	(−8.1047)
LnBed	0.0343*	0.0524*	0.1317***	0.1338**
	(1.9193)	(1.7128)	(7.0003)	(6.9497)
LnEdu	−0.0533*	−0.0369*	0.0260**	0.0318*
	(−1.8437)	(−1.9213)	(1.6946)	(1.9351)
W* LnHI	0.0048	−0.0160	0.0625*	0.0390
	(1.0419)	(−0.9570)	(1.3677)	(0.6509)
W* LnDoc	0.0376	0.0863	0.1324*	0.1027**
	(0.9938)	(1.9092)	(1.8386)	(2.4543)
W* LnBed	−0.0683*	−0.0387	0.0966**	−0.0936
	(1.3488)	(−0.9411)	(1.9616)	(−1.4904)
W* LnEdu	−0.0386	0.1489	−0.1152*	−0.1270**
	(−0.4205)	(1.3573)	(−1.9342)	(−2.0066)
W*dep.var.	0.1199*	0.1339*	0.1340**	0.1099
	(1.5016)	(1.2130)	(1.9292)	(0.9944)
	0.0047	0.0056	0.0366	0.0375
*R* ^2^	0.3577	0.9139	0.6577	0.6361
C*orrected*.*R*^2^	0.3544	0.3591	0.6544	0.5307
LogL	57.8541	303.5458	310.4060	58.9300

***, **, * Represent significance levels of 1, 5, and 10%, respectively.

**Table 6:** Descriptive statistical table of cumulative effect scalars.

**Variable**	**Direct effect**	**T value**	**Indirect effect**	**T value**	**Total effect**	**T value**
LnHI	0.0660***	4.2275	0.0112*	1.5572	0.0772***	1.9983
LnDoc	−0.1058***	−8.4654	−0.0143*	−1.0119	−0.1201***	−6.0495
LnBed	0.1312***	7.0924	0.0576*	2.5033	0.1888***	8.5785
LnEdu	0.0262*	1.6706	0.0038	0.7846	0.0301*	1.6046

***, * Represent significance levels of 1% and 10%, respectively.

LeSage et al. (33) deduced the direct effect of measuring the influence of the change of independent variable on the dependent variable of the neighborhood and the indirect effect of measuring the influence of the change of independent variable on the dependent variable of the neighborhood based on the model's own partial derivative and crosspartial derivative. Elhorst applied the partial differential method to measure direct effect, indirect effect, and total effect (34), with the results shown in Table 6.

Overall, the direct effect coefficient of LnHI is 0.0660, which is significant at 1% significance level. The indirect effect coefficient is 0.0112 and significant. Improving LnHI in local regions can improve their health, and it may improve health improvement in adjacent regions. The total effect is positive and significant, which indicates that LnHI does improve health. This shows that health input improves the health level of residents in the region (14–16). Besides, it has a positive demonstration effect on neighboring provinces to increase health input and health output.

The direct and indirect effects of LnDoc on health are −0.1058 and −0.0143, respectively, which are significant at 1 and 10% confidence level. The significance test reflects that LnDoc has significant spatial spillover effects. Direct effect of LnDoc is greater than indirect effect. The total effect is negative and significant, which is consistent with some scholars' conclusion (13, 25). This shows that increasing the number of health personnel in this region does not promote the health output of this region, which may be because economically developed regions will attract the population of surrounding regions to transfer to areas with rich health resources and economically developed regions, which leads to the uneven regional distribution of health human capital in various regions and seriously affects the exertion of the spatial spillover effect of labor force.

The direct effect coefficient of LnBed is 0.1312, which is significant at 1% confidence level. The indirect effect coefficient is 0.0576, which is significant at 10% confidence level. Improving LnBed promotes health in local and neighboring provinces. The total effect is positive and significant. This phenomenon is not difficult to understand. According to Grossman's model (2), this may be increasing the number of beds which will promote residents' demand for health, so as to improve their health status and improve their health level; besides, it has a positive demonstration effect on neighboring provinces to increase LnBed input.

The direct effect coefficient of LnEdu is 0.0262, which is significant at 10% confidence level. The indirect effect coefficient is 0.0038, which is not significant. Improving LnEdu promotes health in local regions (2, 26). This shows that improving the education level of local residents will play an important role in promoting people to obtain job opportunities, improve the quality of life, form a good lifestyle, and obtain good medical conditions, and the better their health level will be. The total effect is positive and significant. This may be because on the one hand, China's per capita education level is still relatively low compared with developed countries such as Europe and the United States; on the other hand, Chinese college students prefer to work in economically developed cities and coastal areas with high wage levels after graduation, resulting in huge regional differences in education and urban–rural differences.

The spatial autoregressive coefficient on W^*^dep.var is 0.1340, which is significant at 5% confidence level, which indicates that the spatial lag variable has a positive and significant effect on health. That is, the interaction and radiation effect of health output in the 31 provinces are significant, and the positive spillover effect of places with high health level will spread to the surrounding areas and play a positive role in promoting it. Therefore, Chinese provinces should continue to maintain the momentum of increasing health investment, improve the health level of the people, and then promote the common prosperity of other regions. In addition, health policies should not be formulated in isolation, but should take into account the overall situation of the country and develop in coordination with other provinces.

## Conclusions, Recommendations, And Limitations

The above results show that the health output of the 31 provinces in China not only benefits from the local health input, but also benefits from the health input of neighboring provinces. At the same time, due to the significant spatial dependence among different regions, the interaction effect of health output between each province and its neighboring provinces is obvious. Therefore, to give full play to the spatial spillover effect of health input, rationally allocate health resources, and further promote regional economic development, the following suggestions are put forward:

(1) Chinese government should strengthen health collaboration between neighboring provinces and promote the free flow of resources, health professionals, and other elements between provinces. China should strengthen the spillover effect between provinces, give play to the benign interaction of health output, and promote the coordinated development of China's overall health output. Local governments should break the standard ideology, establish the overall awareness, and actively establish the health cooperation system. In the process of implementing health policies, China government should pay attention to overall planning, avoid waste of resources, and take improving China's health output as the primary task of building a healthy China, so as to realize the coordinated development of regional health.
(2) China government should take advantage of the spillover and interprovincial interdependence of health inputs at the provincial level. China should encourage exchanges and cooperation among different regions, actively encourage the flow of health technical personnel, and improve the level of knowledge spillover. At present, China's health resources and health technicians are concentrated in the economically developed coastal areas, whereas there are relatively few in the northwest and border areas. Therefore, attention should be paid to building a central area with diffusion effect, and transregional talent exchange should be used as a link to drive the surrounding areas, so as to realize the balanced development of health levels in different regions. For the coastal economically developed areas, enhance their own radiation effect, the less developed areas should make full use of the spillover effect to make up for the lack of medical technology capacity, save the cost of health research and development, give full play to the advantages of late-comers, and actively connect with the developed areas.
(3) Another important factor that influences knowledge spillover effect is the health policy, so it is necessary to improve the China's health policy environment. It is conducive to the spread of spillover effect of health investment to improve the hard environment of basic supporting service facilities such as hospitals, health centers, and clinics and to establish efficient and reasonable modern medical and health system to narrow the gap of medical technology level between provinces and regions. The most important is to continuously improve the soft environment for health development, accelerate the perfection of relevant laws, improve the open market mechanism, standardize the hospital management system and supervision mechanism, and form a good medical atmosphere.

There are two limitations in the study. First, the models we built are based on the spatial panel data of 31 provinces. But most health management activities take place in cities, whereas the utilization of provincial-level data makes it difficult to capture the spatial heterogeneity of different cities, so the utilization of city-level data is necessary to increase the reliability of estimations. Second, this article simply uses a province's overall health input to measure the impact of health input indicators (HI) on health output, without considering the impact on health output from the perspective of the structural characteristics of health input. In the future, we can measure the impact of health input indicators on health output in terms of government health expenditures, social health expenditures, and personal health expenditures.

## Data Availability Statement

The original contributions presented in the study are included in the article/supplementary material, further inquiries can be directed to the corresponding author/s.

## Author Contributions

All authors listed have made a substantial, direct, and intellectual contribution to the work and approved it for publication.

## Funding

This study was funded by the grants from the Ministry of Education of China (Grant 20YJCZH193) and Anhui Provincial Department of Education (Grant SK2018A0203).

## Conflict of Interest

The authors declare that the research was conducted in the absence of any commercial or financial relationships that could be construed as a potential conflict of interest.

## Publisher's Note

All claims expressed in this article are solely those of the authors and do not necessarily represent those of their affiliated organizations, or those of the publisher, the editors and the reviewers. Any product that may be evaluated in this article, or claim that may be made by its manufacturer, is not guaranteed or endorsed by the publisher.
